# Chemokine Ligand 5 (CCL5) and chemokine receptor (CCR5) genetic variants and prostate cancer risk among men of African Descent: a case-control study

**DOI:** 10.1186/1897-4287-10-16

**Published:** 2012-11-20

**Authors:** LaCreis R Kidd, Dominique Z Jones, Erica N Rogers, Nayla C Kidd, Sydney Beache, James E Rudd, Camille Ragin, Maria Jackson, Norma McFarlane-Anderson, Marshall Tulloch-Reid, Seian Morrison, Guy N Brock, Shirish S Barve, Kevin S Kimbro

**Affiliations:** 1 Department of Pharmacology & Toxicology, University of Louisville Clinical Translational Research Building, 505 South Hancock Street Room 306, Louisville, KY, 40202, USA; 2 Biomedical/Biotechnology Research Institute (BBRI), North Carolina Central University, 531 Nelson Street, Durham, NC, 27707, USA; 3 Cancer Prevention and Control Program, Fox Chase Cancer Center, 333 Cottman Avenue, Philadelphia, PA, 19111, USA; 4 Department of Community Health and Psychiatry, University of West Indies, Ring Road, Mona, Jamaica; 5 Department of Bioinformatics & Biostatistics, University of Louisville, School of Public Health & Information Sciences, 485 East Gray Street, Louisville, KY, 40202, USA; 6 School of Medicine, University of Louisville, 323 East Chestnut Street, Louisville, KY, 40292, USA

**Keywords:** Prostate cancer, Chemokines, Chemokine receptors, Chemokine ligand 5, Chemokine receptor 5, Single nucleotide polymorphisms, Men of African descent

## Abstract

**Background:**

Chemokine and chemokine receptors play an essential role in tumorigenesis. Although chemokine-associated single nucleotide polymorphisms (SNPs) are associated with various cancers, their impact on prostate cancer (PCA) among men of African descent is unknown. Consequently, this study evaluated 43 chemokine-associated SNPs in relation to PCA risk. We hypothesized inheritance of variant chemokine-associated alleles may lead to alterations in PCA susceptibility, presumably due to variations in antitumor immune responses.

**Methods:**

Sequence variants were evaluated in germ-line DNA samples from 814 African-American and Jamaican men (279 PCA cases and 535 controls) using Illumina’s Goldengate genotyping system.

**Results:**

Inheritance of *CCL5* rs2107538 (AA, GA+AA) and rs3817655 (AA, AG, AG+AA) genotypes were linked with a 34-48% reduction in PCA risk. Additionally, the recessive and dominant models for *CCR5* rs1799988 and *CCR7* rs3136685 were associated with a 1.52-1.73 fold increase in PCA risk. Upon stratification, only *CCL5* rs3817655 and *CCR7* rs3136685 remained significant for the Jamaican and U.S. subgroups, respectively.

**Conclusions:**

In summary, *CCL5* (rs2107538, rs3817655) and *CCR5* (rs1799988) sequence variants significantly modified PCA susceptibility among men of African descent, even after adjusting for age and multiple comparisons. Our findings are only suggestive and require further evaluation and validation in relation to prostate cancer risk and ultimately disease progression, biochemical/disease recurrence and mortality in larger high-risk subgroups. Such efforts will help to identify genetic markers capable of explaining disproportionately high prostate cancer incidence, mortality, and morbidity rates among men of African descent.

## Background

Prostate cancer (PCA) is one of the leading causes of cancer and cancer-related deaths among men in the U.S. and Caribbean and its disparities are well documented [1-3]. From 2003–2007, the average annual PCA incidence rates (229.4 per 100,000) and mortality (54.2 per 100,000) rates among African-American men were 1.6- and 2.4-fold higher than US Caucasian men, respectively. According to the 2008 World age-standardized death rates for Caribbean men (26.3/100,000) were 2.65-fold higher than all U.S. men (9.9/100,000) [3]. Despite its public health burden, the etiology of PCA is not completely elucidated. Established risk factors for PCA include older age, black race, and family history of the disease. Other plausible contributors of prostate tumorigenesis include endocrine, lifestyle, environmental, and genetic factors as well as imbalances in inflammatory, immune surveillance, and angiogenesis pathways.

Among the inflammatory factors, chemokines (e.g., CC, CXC, XCL, and C-X3-C) play a pivotal role in chemotaxis, leukocyte trafficking, lymphocyte development, angiogenesis, host response to infection, inflammatory processes, tumor development, migration and metastasis. Chemokines mediate their actions through 7-transmembrane, G protein coupled receptors and serve three major physiological functions. First, they play fundamental roles in the maturation, homeostasis and function of the immune system, as well as facilitate the trafficking of memory T cells, lymphocytes, monocytes, and neutrophils to the inflammatory site. Secondly, they display chemotactic activity for lymphocytes, monocytes, and neutrophils. Lastly, they attract cancer cells and chemokine receptor bearing cells and have effects on endothelial cells involved in angiogenesis regulation. Several CXC chemokines are potent angiogenesis promoters (i.e., CXCL1, 2, 3, 5, 6, 7) [4-6]; whereas, others inhibit angiogenesis (i.e., CXCL4, 9, 10, 11) [7].

Genetic alterations and altered expression of chemokines and their receptors have been linked to the susceptibility, development and survival of numerous cancers, including prostate cancer [8-12]. In fact, over expression of *CCL5* and *CCR5* has been detected in prostate tissue and associated with aggressive disease, presumably by triggering leukocyte production and promoting cell survival, proliferation, invasion and metastasis [13-15]. Coding and regulatory regions of a number of chemokine-associated genes directly influence chemokine production and have been demonstrated to modulate the risk of developing various cancers [10,16-21]. The *CCL5* -403A allele conferred an increased risk of prostate, oral and pancreatic cancer [18,22,23]. Furthermore, inheritance of the *CCR7* rs3136685 AG+AA or *CCR7* rs3136687 (AG, AG+AA) genotypes was associated with a 60-62% reduction in multiple myeloma (MM) and chronic lymphocytic leukemia, respectively [19,21]. Unfortunately, these previously mentioned studies focused on single SNPs in relation to cancer primarily among men of European descent. Consequently, the influence of individual *CCL5*, *CCR5*, *CCR7* and other chemokine associated sequence variants on PCA is unknown among men of African descent.

Thus, the current study evaluated main effects of 43 chemokine-related sequence variants in relation to prostate cancer susceptibility among men of African Descent from the U.S. and Jamaica. Preliminary findings from this study will guide future studies that seek to find important diagnostic, tumor classification and prognostication indicators of PCA in high-risk subgroups.

## Methods

### Study population

The current study consisted of 279 cases and 535 controls obtained from two independent case control study sets, as summarized in Table 1, Additional file 1, and Additional file 2. These studies include the Prostate Cancer Clinical Outcome Study (PC^2^OS) at the University of Louisville and the Prostate Cancer Study in Jamaica at the University of the West Indies, Mona Campus. For the PC^2^OS study, 603 unrelated male residents were recruited from the Washington, D.C. and Columbia, SC areas through the Howard University Hospital (HUH) Division of Urology or related PCA screening programs between 2001 and 2005. This population of men of African descent (i.e., self-reported African Americans, East African Americans, West African Americans, and Afro-Caribbean Americans) consisted of 170 incident PCA cases and 433 controls (Additional file 1). Between March 2005 and July 2007, two hundred twenty-one unrelated Jamaican men were recruited and consecutively enrolled into the prostate cancer case–control study (109 prostate cancer cases, 102 controls) during their first time visit at urology clinics (Additional file 2). Details on case and control ascertainment and inclusion criteria for both sub-populations have ben detailed elsewhere [24,25]. All study participants provided written informed consent for participation in genetic analysis studies under a protocol approved by the Howard University, the HUH Division of Urology, and the University of Louisville Institutional Review Board (#201.07).

**Table 1:** Baseline Characteristics among men of African Descent from the US & Jamaica

**Characteristics**	**Cases**	**Controls**	**p value**^**†**^
Number of Participants, n	279	535	---
Age at enrollment (yrs), Median (range)	67 (45–91)	53 (27–89)	<0.0001
Family History of Prostate Cancer, n (%)			
Yes	35 (16.1)	21 (12.5)	0.316
No	182 (83.9)	147 (87.5)	
Missing	62 (22.2)	367 (68.6)	
PSA (ng/ml), median	11.7 (0.01-10,000)	0.9 (0.0-4.0)	<0.0001
PSA (ng/ml), n (%)			
< 4	37 (13.8)	517 (99.8)	<0.0001
≥ 4	231 (86.2)	1 (0.2)	
Missing	11 (0.04)	17 (0.03)	
Gleason Score, n (%)			
4	12 (5.6)		
5	14 (6.5)		
6	74 (34.2)		
7	70 (32.4)		
8	18 (8.3)		
9	22 (10.2)		
10	6 (2.8)		
Missing	63 (22.6)		
Global WAA, mean (SD)	0.79 (0.25-0.94)	0.767 (0.25-0.95)	0.107

Abbreviations: PSA, prostate specific antigen; WAA, West African Ancestry; ^†^Differences in family history or PSA frequencies between cases and controls were tested by a Chi-square test of homogeneity; differences in median age (yrs) and Global West African Ancestry between cases and controls were tested using the Wilcoxon sum Rank test.

### Criteria for chemokine and chemokine receptor gene and SNP selection

Chemokine-associated genes and SNPs were selected using one or more of following criteria: (1) epidemiological or molecular biological evidence from published reports indicating a relationship between the SNP/gene with cancer or inflammatory/immune response related diseases; (2) commonly studied loci; (3) marked disparities in genotype frequency comparing men of African descent to their Caucasian counterparts (i.e., ±10% change); (4) evidence demonstrating a link with alterations in mRNA expression/stability or protein expression/structure or function using *in silico* tools (e.g., SNPinfo) or published reports (http://snpinfo.niehs.nih.gov/snpfunc.htm); and (5) a minor allele frequency ≥1% reported in the National Center for Biotechnology Information (NCBI) Entrez SNP, (http://www.ncbi.nlm.nih.gov). On average, a majority of the SNPs had minor allele frequencies ranging from 26-27%. However, five SNPs with allele frequencies greater than 1% but less than 5% were included in the analysis to explore whether rare SNPs would lead to substantial gains in effect sizes (i.e., 2–3 fold increases in risk) and contribute to missing genetic heritability [26,27]. The SNPinfo webserver enabled us to annotate and/or predict the functional consequence of chemokine-associated sequence variants on alternative alleles, as summarized in Additional file 3.

### Genetic analysis of variant chemokine-associated SNPs

In order to evaluate and validate chemokine-related markers as predictors of PCA risk, de-identified germ-line DNA from PCA cases and disease-free individuals were genotyped using a custom Illumina GoldenGate Genotyping assay with VeraCode Technology and BeadXpress reader, according to the manufacturer’s instructions [28].

### Ancestry markers

Cases and controls were also genotyped with a set of 100 genome-wide ancestry informative markers to correct for potential population stratification among our admixed population of self-reported African-Americans, West African-Americans, East African-Americans, Afro-Caribbean-Americans, as previously described [29,30]. Study participants were grouped from lowest to highest genetic West African Ancestry, with scores ranging from 0-100%. These 100 markers were assembled using DNA from self-identified African-Americans (Coriell Institute for Medical Research, n = 96), Yoruban West Africans (HapMap, n = 60), West Africans (Bantu and Nilo Saharan speakers, n = 72), Europeans (New York City, n = 24), and CEPH Europeans (HapMap Panel, n = 60), as previously reported [30]. Individuals with a West African ancestry (WAA) score ≥25% and available chemokine-associated data were included in the final analysis.

### Quality control assurance and data management of genotype data

At the onset of the project, allelic discrimination focused on chemokine associated SNPs among men of African Descent. To minimize misclassification bias, laboratory technicians were blinded to the case status of study participants. Each batch of up to 96 samples included four non-DNA template controls and eight duplicate samples, enabling us to calculate the percent contamination (i.e., 0%) and concordance rates (i.e., 100%) per batch. Genotype call rates were calculated separately for each SNP and study participant. Lastly, we tested whether the distribution of the genotypes among disease-free individuals had significant departures from the Hardy-Weinberg equilibrium (HWE).

Prior to performing marker statistics, we excluded subjects who had genotype call rates that were <90%. To ensure high quality data, nine SNPs were excluded from the final analysis if: the distribution of the genotypes among controls deviated substantially from the Hardy-Weinberg Equilibrium, using a conservative significance level cut-off value of P ≤ 0.005 (n = 1); they had a minor allele frequency <1% (n = 6); or low genotype call rates <95% (n = 2). Following data clean-up, 43 chemokine-related SNPs were included in the final analysis consisting of 814 men of African descent (279 cases, 535 controls). All quality control analyses and data management was performed using Golden Helix’s SNP Variation Software 7.0 (Bozeman, MT).

### Statistical analysis for single gene effects

Univariate and multivariate analyses were used to evaluate chemokine-associated SNPs among men of African descent in relation to prostate cancer risk. To assess whether inheritance of one or more chemokine allele(s) influence the risk of developing PCA, we tested for significant differences in the distribution of homozygous major, heterozygous, or homozygous minor genotypes between cases and controls using the chi-square test of homogeneity. The association between PCA risk and selected polymorphic genes, expressed as odds ratios (ORs) and corresponding 95% confidence intervals (CIs), were estimated using unconditional multivariate LR models adjusted for age. LR analyses for genetic variants and PCA development were conducted using the major or common genotype as the referent category. All analyses were conducted using SAS 9.3 (SAS Institute Inc., Cary, NC) and SNP Variation Software 7.0 (GoldenHelix, Bozeman, MT). Statistical significance was assessed using a False Discovery Rate cut-off of 0.05, in order to adjust for multiple comparisons.

### Statistical power for single gene effects

We conducted calculations to determine the statistical power of our sample to detect significant relationships between chemokine-related genotypes and PCA development. The expected risk estimates of our study can be estimated by specifying values for a number of parameters, including a minor allele frequency of at least 26.5%, National Cancer Institute's estimate of PCA disease prevalence (0.74%), number of cases (n = 279), and number of controls (n = 535). We assumed the causal SNP was in complete linkage disequilibrium with the predisposing variant (r^2^ = 1.0). Based on our sample size for the total population, U.S. and Jamaican men, we had >80% power to detect genetic markers with odds ratios (ORs) of ≥1.5, ≥1.55, and ≥2.0 for PCA risk, respectively, for minor allele frequency of at least 26.5%, assuming a co-dominant genetic model with 1 degree of freedom (df). Power calculations were performed using Power for Genetic Association Version 2 Software [31].

## Results

### Population description

The demographic and other pertinent characteristics of cases and controls for the entire study population and each study center are summarized in Table 1, Additional file 1, and Additional file 2. Overall, men diagnosed with prostate cancer were 14 years older and had higher PSA levels than controls (P < 0.0001). Disease-free men from Jamaica were about 9 years older than U.S. controls; however, there were no significant differences in family history of prostate cancer or PSA levels for these disease-free subgroups. Jamaican cases had 7 fold higher PSA levels (P < 0.0001) and slightly higher median Gleason scores (P = 0.018) compared to cases from the U.S. There were no differences in the distribution of family history of prostate cancer comparing: (1) cases to controls from the total population (P = 0.316) (Table 1), U.S. alone (P = 0.592) (Additional file 1), or Jamaica alone (P = 0.272) (Additional file 2); and (2) controls (P = 0.757) or cases (P = 0.830) comparing the two study centers (data not shown).

### Prevalence of minor alleles/genotype frequency comparing men of African Descent from the U.S. and Jamaica

Overall, the chemokine-related SNPs were fairly common among disease-free individuals from the entire sub-population of U.S. and Jamaica, with average minor frequencies of 26-27% and a standard deviation of 14%, respectively (data not shown). Thirty-eight SNPs had minor allele frequencies ≥5%. For exploratory purposes, five rare SNPs (*CCR9* rs12721497, *CCL17* rs11076191, *CCL11* rs4795896, *CCL21* rs11574916, *CXCL12* rs1801157) were analyzed with minor allele frequencies ranging between 1.5-4.9%. The minor allele frequency comparing controls from the U.S. and Jamaica were strongly correlated (R^2^ = 0.957). Only 5 out of the 43 SNPs analyzed were discordant comparing men of African descent from the U.S. to men from Jamaica (P < 0.0487), namely *CCL17* rs11076191, *CCL21* rs11574916, *CCR7* rs3136685, *CCR7* rs3136687, and *CCR9* rs12721497.

### Relationship between chemokine sequence variants and prostate cancer risk

Among all men of African descent, five sequence variants were significantly associated with the risk of developing prostate cancer, as summarized in Table 2. Possession of the *CCL5* rs2107538 GA+AA (OR_unadjusted_ = 0.59; 95%CI = 0.44, 0.80) or *CCL5* rs3817655 GA+AA (OR_unadjusted_ = 0.54; 95%CI = 0.40, 0.74) genotype was linked with a 41-46% reduction in PCA risk in the unadjusted LR models. These effects remained significant for both SNPs after adjusting for age (CCL5 rs2107538: OR_adjusted_ = 0.66; 95%CI = 0.46, 0.96) and *CCL5* rs3817655: OR_adjusted_ = 0.56; 95%CI = 0.39, 0.81). The recessive genetic model for *CCR5* rs1799988 (OR_adjusted_ = 1.52; 95%CI = 1.02, 2.26) as well as the dominant genetic models for *CCR7* rs3136685 (OR_adjusted_ = 1.66; 95%CI = 1.09, 2.54) and *CCR7* rs3136687 (OR_adjusted_ =1.14; 95%CI = 1.12, 1.16) were associated with a significant 1.14-1.66 fold increase in PCA risk within the age adjusted LR models. After controlling for multiple comparisons, the dominant genetic models for the two *CCL5* SNPs (rs2107538, rs3817655) remained significant with false-discovery rates (FDR) ≤ 0.0150; whereas, the recessive genetic model for *CCR5* rs1799988 was marginally significant (FDR = 0.0494).

**Table 2:** Association between chemokine associated SNPs and prostate cancer risk among men of African descent

**Genes**	**dbSNP ID**	**Location**	**Genotype**	**Cases n (%)**	**Controls n (%)**	**Unadjusted**	**Adjusted**	**P-value**	**P-trend**	**FDR**
		**predicted function†**				**OR(95%CI)**	**OR (95%CI)**			
CCR5	rs1799988	5’UTR	AA	85 (30.7)	194 (36.7)	1.00 (referent)	1.00 (referent)	**0**.**005**	**0**.**0039**	0.0682
		TFBS	AG	107 (38.6)	227 (43.9)	1.08 (0.76, 1.52)	0.83 (0.55, 1.25)	0.676		
			GG	85 (30.7)	108 (20.4)	**1**.**80** (**1**.**23**, **2**.**63**)	1.38 (0.87, 2.17)	**0**.**003**		
			AG+GG	192 (69.3)	335 (63.3)	1.31 (0.96, 1.78)	1.01 (0.70, 1.46)	0.09		0.4823
			GG vs (AA+AG)			**1**.**73** (**1**.**24**, **2**.**40**)	**1**.**52** (**1**.**02**, **2**.**26**)	**0**.**0013**		**0**.**0494**
CCL5	rs2107538	5' near gene	GG	111 (39.8)	150 (28.1)	1.00 (referent)	1.00 (referent)	**0**.**002**	**0**.**001**	**0**.**0493**
		TFBS	GA	124 (44.4)	270 (50.6)	**0**.**62** (**0**.**45**, **0**.**86**)	0.72 (0.49, 1.06)	**0**.**004**		
			AA	44 (15.8)	114 (21.4)	**0**.**52** (**0**.**34**, **0**.**80**)	**0**.**53** (**0**.**32**, **0**.**89**)	**0**.**003**		
			GA+AA	168 (60.2)	384 (71.9)	**0**.**59** (**0**.**44**, **0**.**80**)	**0**.**66** (**0**.**46**, **0**.**96**)	**0**.**0007**		0.015
			AA vs (GG+GA)			0.83 (0.57, 1.20)	0.74 (0.47, 1.16)	0.057		0.4735
CCL5	rs3817655	Intron 2	GG	114 (41.0)	147 (27.5)	1.00 (referent)	1.00 (referent)	**0**.**004**	**0**.**002**	**0**.**019**
		TFBS	GA	115 (41.4)	278 (52.0)	**0**.**53** (**0**.**38**, **0**.**74**)	**0**.**57** (**0**.**38**, **0**.**84**)	**0**.**0002**		
			AA	49 (17.6)	110 (20.5)	**0**.**57** (**0**.**38**, **0**.**87**)	**0**.**54** (**0**.**32**, **0**.**89**)	**0**.**0009**		
			GA+AA	164 (59.0)	388 (72.5)	**0**.**54** (**0**.**40**, **0**.**74**)	**0**.**56** (**0**.**39**, **0**.**81**)	**0**.**0001**		**0**.**0038**
			AA vs (GG+GA)			0.83 (0.57, 1.20)	0.74 (0.47, 1.16)	0.317		1
*CCR7*	rs3136685	Intron 1	AA	55 (19.7)	151 (28.3)	1.00 (referent)	1.00 (referent)	**0**.**029**	**0**.**031**	*0.3078*
			AG	139 (49.8)	237 (44.4)	**1**.**61** (**1**.**11**, **2**.**34**)	**1**.**86** (**1**.**18**, **2**.**92**)	0.012		
			GG	85 (30.5)	146 (27.3)	**1**.**60** (**1**.**06**, **2**.**40**)	1.39 (0.85, 2.28)	**0**.**024**		
			AG+GG	224 (80.3)	383 (71.7)	**1**.**61** (**1**.**13**, **2**.**28**)	**1**.**66** (**1**.**09**, **2**.**54**)	**0**.**008**		*0.599*
			GG vs (AA+AG)			1.16 (0.85, 1.60)	0.92 (0.62, 1.35)	**0**.**348**		*0.1615*
*CCR7*	rs3136687	Intron 1	AA	84 (30.1)	173 (32.4)	1.00 (referent)	1.00 (referent)	**0**.**041**	0.458	*0.3523*
			AG	153 (54.8)	249 (46.5)	1.26 (0.91, 1.76)	1.45 (0.97, 2.16)	0.161		
			GG	42 (15.1)	113 (21.1)	0.77 (0.49, 1.19)	0.96 (0.57, 1.62)	0.234		
			AG+GG	195 (69.9)	362 (67.6)	1.11 (0.81, 1.52)	**1**.**14** (**1**.**12**, **1**.**16**)	0.516		0.7159
			GG vs (AA+AG)			**0**.**66** (**0**.**45**, **0**.**98**)	0.76 (0.49, 1.20)	**0**.**037**		0.5092

In an exploratory analysis, we evaluated risk estimates for all 43 chemokine targets for each racial/ethnic group, as depicted in Table 3. Among U.S. men, CCR5 rs1799988, *CCL5* (rs2107538, rs2280789, rs3817655), *CCL25* rs2032887, and *CXCR7* rs1045879 were associated with PCA risk. Inheritance of the *CCL25* rs2032887 AG+GG (OR_unadjusted_ = 0.66; 95%CI = 0.46, 0.96), *CCL5* rs2107538 GA+AA (OR_unadjusted_ = 0.52; 95%CI = 0.36, 0.76), *CCL5* rs2280789 AG+GG (OR_unadjusted_ = 0.60; 95%CI = 0.41, 0.89), and *CCL5* rs3817655 GA+AA (OR_unadjusted_ = 0.46; 95%CI = 0.32, 0.68) genotypes were significantly associated with a 34-54% reduction in the risk of developing PCA with chi-square p-values ranging from 0.0001-0.027. Although the magnitude of the reduction in PCA risk for *CCL5* rs2107538 GA+AA (OR_unadjusted_ = 0.52; OR_adjusted_ = 0.63), *CCL5* rs3817655 GA+AA (OR_unadjusted_ = 0.46, OR_adjusted_ = 0.51) and *CCL25* rs2032887 AG+GG (OR_unadjusted_ = 0.66, OR_adjusted_ = 0.68) genotypes remained practically unchanged after adjusting for age, the findings only remained significant for *CCL5* rs33817655 SNP after the adjustment. The 1.54-1.62 fold increase in PCA susceptibility was associated with the *CCR5* rs1799988 recessive genetic model (OR_unadjusted_ =1.62; 95%CI=1.08, 2.42) and the *CXCR7* rs1045879 AG+GG genotype (OR_unadjusted_ = 1.54; 95%CI = 1.07, 2.22; P = 0.02) was lost in the age adjusted risk models.

**Table 3:** Association between chemokine associated SNPs and prostate cancer risk, stratified by racial ethnic group

**Genes**	**dbSNP ID**	**Location**	**Genotype**	**Unadjusted**	**Unadjusted**	**Age-adjusted**	**Age-adjusted**	**P-value**	**P trend**	**P-vlue**	**P-trend**
		**predicted function**		**OR(95%CI)**	**OR(95%CI)**	**OR(95%CI)**	**OR(95%CI)**	**US men**	**US men**	**Jamaican men**	**Jamaican men**
				**US men**	**Jamaican men**	**US men**	**Jamaican men**				
*CCR5*	rs1799988	5’UTR	AA	1.00 (referent)	1.00 (referent)	1.00 (referent)	1.00 (referent)	0.063	0.076	0.085	**0**.**034**
		TFBS	AG	0.95 (0.62, 1.44)	1.26 (0.66, 2.40)	0.68 (0.42, 1.12)	1.23 (0.60, 2.52)	0.808		0.484	
			GG	1.58 (0.99, 2.49)	**2**.**25** (**1**.**08**, **4**.**71**)	1.06 (0.60, 1.85)	2.23 (0.99, 5.00)	0.053		**0.031**	
			AG+GG	1.15 (0.79, 1.68)	1.56 (0.86, 2.82)	0.80 (0.52, 1.26)	1.55 (0.81, 2.98)	0.453		0.142	
			GG vs (AA+AG)	**1**.**62** (**1**.**08**, **2**.**42**)	**1**.**96** (**1**.**04**, **3**.**70**)	1.28 (0.78, 2.12)	1.98 (0.98, 3.98)	**0**.**02**		**0**.**037**	
*CCL5*	rs2107538	5' near gene	GG	1.00 (referent)	1.00 (referent)	1.00 (referent)	1.00 (referent)	**0**.**003**	**0**.**002**	0.286	0.116
		TFBS	GA	**0**.**54** (**0**.**36**, **0**.**80**)	0.75 (0.40, 1.40)	0.67 (0.42, 1.08)	0.82 (0.40, 1.64)	**0**.**003**		0.375	
			AA	**0**.**48** (**0**.**28**, **0**.**82**)	0.53 (0.24, 1.16)	0.53 (0.28, 1.01)	0.52 (0.22, 1.22)	**0**.**007**		0.116	
			AG+AA	**0**.**52** (**0**.**36**, **0**.**76**)	0.68 (0.38, 1.23)	**0**.**63** (**0**.**40**, **0**.**99**)	0.72 (0.37, 1.40)	**0**.**001**		0.204	
			AA vs (GG+GA)	0.68 (0.42, 1.11)	0.64 (0.32, 1.26)	0.66 (0.37, 1.20)	0.58 (0.28, 1.24)	0.125		0.192	
*CCL5*	rs2280789	Intron 1	AA	1.00 (referent)	1.00 (referent)	1.00 (referent)	1.00 (referent)	**0**.**039**	**0**.**018**	0.508	0.823
		TFBS	AG	**0**.**60** (**0**.**40**, **0**.**90**)	1.20 (0.66, 2.18)	**0**.**60** (**0**.**37**, **0**.**97**)	1.48 (0.76, 2.89)	**0**.**015**		0.549	
			GG	0.62 (0.24, 1.56)	0.59 (0.18, 1.89)	1.00 (0.34, 2.99)	0.42 (0.12, 1.46)	**0**.**309**		0.373	
			AG+GG	**0**.**60** (**0**.**41**, **0**.**89**)	1.06 (0.61, 1.86)	0.64 (0.40, 1.01)	1.18 (0.63, 2.20)	**0**.**011**		0.82	
			GG vs (AA+AG)	0.72 (0.28, 1.82)	0.55 (0.18, 1.80)	1.14 (1.12, 1.18)	0.36 (0.10, 1.27)	0.488		0.313	
*CCL5*	rs3817655	Intron 2	GG	1.00 (referent)	1.00 (referent)	1.00 (referent)	1.00 (referent)	**0**	**0**.**003**	0.275	0.11
		TFBS	GA	**0**.**44** (**0**.**29**, **0**.**65**)	0.72 (0.38, 1.34)	**0**.**49** (**0**.**30**, **0**.**80**)	0.74 (0.36, 1.50)	<**0**.**0001**		0.302	
			AA	**0**.**56** (**0**.**34**, **0**.**92**)	0.53 (0.24, 1.16)	0.54 (0.29, 1.02)	0.50 (0.22, 1.20)	**0**.**022**		0.115	
			GA+AA	**0**.**46** (**0**.**32**, **0**.**68**)	0.66 (0.36, 1.20)	**0**.**51** (**0**.**32**, **0**.**80**)	0.66 (0.34, 1.28)	<**0**.**0001**		0.167	
			AA vs (GG+GA)	0.88 (0.55, 1.40)	0.66 (0.34, 1.28)	0.80 (0.46, 1.42)	0.61 (0.28, 1.27)	0.577		0.22	
*CCL25*	rs2032887	Exon 3	AA	1.00 (referent)	1.00 (referent)	1.00 (referent)	1.00 (referent)	0.084	**0**.**034**	0.474	0.379
		ESE or ESS	AG	**0**.**67** (**0**.**46**, **0**.**99**)	1.42 (0.80, 2.48)	0.70 (0.44, 1.11)	1.54 (0.82, 2.88)	0.404		0.225	
		nsSNP	GG	0.61 (0.29, 1.28)	1.10 (0.36, 3.38)	0.57 (0.22, 1.42)	0.84 (0.24, 2.88)	0.196		0.86	
		probably damaging	AG+GG	**0**.**66**, (**0**.**46**, **0**.**96**)	1.37 (0.80, 2.36)	0.68 (0.44, 1.05)	1.42 (0.78, 2.60)	**0**.**027**		0.253	
		missense R>H	GG vs (AA+AG)	0.71 (0.34, 1.47)	0.93 (0.32, 2.75)	0.65 (0.26, 1.60)	0.68 (0.20, 2.24)	0.357		0.898	
*CCR5*	rs1799987	Intron 1	GG	1.00 (referent)	1.00 (referent)	1.00 (referent)	1.00 (referent)	0.197	0.951	0.107	**0**.**041**
		TFBS	GA	1.29 (0.88, 1.92)	1.28 (0.68, 2.44)	0.89 (0.56, 1.44)	1.25 (0.61, 2.56)	0.191		0.442	
			AA	0.84 (0.48, 1.48)	**2**.**18** (**1**.**04**, **4**.**58**)	0.58 (0.29, 1.14)	2.20 (0.98, 4.94)	0.558		**0**.**039**	
			GA+AA	1.18 (0.80, 1.70)	1.56 (0.86, 2.82)	0.81 (0.52, 1.26)	1.55 (0.81, 2.98)	0.399		0.142	
			AA vs (GG+GA)	0.72 (0.44, 1.21)	1.88 (0.99, 3.56)	0.61 (0.32, 1.15)	1.92 (0.96, 3.88)	0.222		0.051	
*CCR7*	rs3136685	Intron 1	AA	1.00 (referent)	1.00 (referent)	1.00 (referent)	1.00 (referent)	0.381	0.731	0.103	0.086
			AG	1.32 (0.86, 2.02)	2.24 (0.98, 5.16)	1.58 (0.94, 2.66)	**2.78 (1.09, 7.08)**	0.197		0.056	
			GG	1.07 (0.65, 1.76)	**2.38 (1.02, 5.58)**	1.02 (0.56, 1.86)	2.52 (0.97, 6.52)	0.778		**0**.**045**	
			AG+GG	1.23 (0.82, 1.84)	**2**.**3** (**1**.**05**, **5**.**07**)	1.36 (0.83, 2.21)	**2**.**66** (**1**.**10**, **6**.**42**)	0.305		**0**.**037**	
			GG vs (AA+AG)	0.90 (0.59, 1.36)	1.28 (0.74, 2.25)	0.76 (0.46, 1.26)	1.16 (0.62, 2.15)	0.622		0.372	
*CCR7*	rs3136687	Intron 1	AA	1.00 (referent)	1.00 (referent)	1.00 (referent)	1.00 (referent)	**0**.**022**	0.294	0.229	0.218
			AG	1.42 (0.89, 2.24)	2.16 (0.84, 5.56)	1.47 (0.85, 2.54)	1.52 (0.53, 4.38)	0.133		0.11	
			GG	0.78 (0.46, 1.34)	2.20 (0.84, 5.69)	0.85 (0.45, 1.59)	1.34 (0.46, 3.96)	0.38		0.106	
			AG+GG	1.17 (0.76, 1.81)	2.18 (0.88, 5.38)	1.22 (0.72, 2.05)	1.44 (0.52, 3.98)	0.482		0.092	
			GG vs (AA+AG)	0.61 (0.40, 0.93)	1.17 (0.68, 2.02)	1.14 (1.12, 1.18)	0.94 (0.51, 1.75)	**0**.**023**		0.573	
*CCR9*	rs1488371	Intron 2	CC	1.00 (referent)	1.00 (referent)	1.00 (referent)	1.00 (referent)	**0**.**013**	0.902	**0**.**003**	**0**.**018**
			CA	0.94 (0.62, 1.40)	0.52 (0.26, 1.08)	0.88 (0.54, 1.42)	0.60 (0.26, 1.31)	0.747		0.08	
			AA	1.57 (0.50, 4.90)	---	0.92 (0.22, 3.80)	---	0.435		0.984	
			CA+AA	0.98 (0.66, 1.44)	**0**.**46** (**0**.**23**, **0**.**94**)	0.88 (0.54, 1.40)	0.48 (0.22, 1.04)	0.901		**0**.**034**	
			AA vs (CC + CA)	1.60 (0.52, 4.97)	---	0.95 (0.23, 4.00)	---	0.414		0.984	
*CXCR7*	rs1045879	Exon 1	AA	1.00 (referent)	1.00 (referent)	1.00 (referent)	1.00 (referent)	0.065	**0**.**038**	0.459	0.762
		synSNP	AG	**1**.**54** (**1**.**06**, **2**.**26**)	1.38 (0.77, 2.48)	1.30 (0.82, 2.04)	1.30 (0.68, 2.50)	**0**.**025**		0.279	
		L>L	GG	1.53 (0.84, 2.79)	0.86 (0.34, 2.16)	1.02 (0.47, 2.20)	0.74 (0.27, 2.04)	0.166		0.756	
			AG+GG	**1**.**54** (**1**.**07**, **2**.**22**)	1.24 (0.72, 2.14)	1.24 (0.80, 1.93)	1.15 (0.62, 2.10)	**0**.**02**		0.431	
			GG vs (AA+AG)	1.21 (0.69, 2.13)	0.76 (0.31, 1.84)	0.88 (0.42, 1.84)	0.67 (0.25, 1.80)	0.499		0.538	

Abbreviations: UTR, untranslated region; TFBS, transcription factor binding site; ESE, exonic splicing enhancers ; ESS, exonic splicing silencers; nsSNP, non-synonymous coding SNP; synSNP, synonymous SNP.

In the Jamaican population, there was a two-fold increase in PCA susceptibility associated with the *CCR5* rs1799987 AA (OR_unadjusted_ = 2.18; 95% CI=1.04, 4.58), *CCR5* rs1799988 GG (OR_unadjusted_ =2.25; 95% CI = 1.08, 4.71), and *CCR7* rs3136685 AG+GG (OR_unadjusted_ = 2.38; 95% CI = 1.05, 5.07) genotypes, with corresponding chi-square P-values ranging from 0.02-0.037. Additionally, a 54% reduction in PCA risk was observed for individuals who possessed the *CCR9* rs1488371 CA+AA genotype (OR_unadjusted_ = 0.46; 95% CI = 0.23, 0.94). Out of the 4 markers, the *CCR7* rs3136685 SNP remained significant after adjusting for age. Notably, the magnitude of PCA risk estimates did not change for the *CCR5*, *CCL5*, and *CCR9* SNPs, comparing the adjusted and unadjusted risk models.

## Discussion

Chemotaxis is an important process required for tumor growth and metastasis. It is regulated by a complex network of chemokines, chemokine receptors and downstream targets that synergistically regulate immune and inflammatory responses. Recent molecular studies have demonstrated that over expression of selected chemokines and chemokine receptors are related to aggressive cancer phenotypes, including lung, breast and prostate cancer. Some observational studies suggest inheritance of susceptibilities detected in chemokine associated genes may alter the risk of developing cancer. However, to our knowledge, there are no published reports on the impact of inheriting multiple functional variants in relation to prostate cancer among men of African Descent. Therefore, the current study evaluated the individual effects of 43 chemokine associated sequence variants on PCA risk among 279 cases and 535 disease-free men of African descent from the U.S and Jamaica. Five SNPs detected in *CCL5*, *CCR5* and *CCR7* were significantly associated with prostate cancer risk among all study participants; however, only three markers survived adjustments for potential confounders and multiple hypothesis testing. Notably, inheritance of at least one *CCL5* rs3817655 A or *CCL5* rs2107538 A loci was linked with a 34-44% decrease in PCA susceptibility among all men of African Descent. In addition, the recessive genetic model for *CCR5* rs1799988 was associated with a 52-73% increase in PCA risk. We also observed significant main effects for the *CCL5* rs3817655 and *CCR7* rs3136685 SNPs among U.S. and Jamaican men, respectively. Further discussion will be restricted to the more robust SNPs detected in *CCL5* and *CCR5*.

Several cancer cells, including PCA cells, express chemokines and their cell surface bound receptors. Chemokine ligand 5 (RANTES), is a small molecule with a strong capacity to induce cellular migration of inflammatory cells and production of its receptor (CCR5) in human PCA cell lines [13,32]. The CCL5/CCR5 axis induces PCA cell proliferation and cell invasion. It’s speculated that once CCL5 binds to CCR5, it serves as an autocrine factor and activates cellular responses involved in cancer progression [13]. In the current study, possession of the *CCL5* rs3817655 A or *CCL5* rs2107538 A loci was linked with a protective effect in relation to prostate cancer risk among all men of African descent from the U.S. and Jamaica combined. The directionality of the risk estimates persisted when we stratified by racial/ethnic group; however, the findings were only statistically significant for the U.S. men. In addition, findings for the total and U.S. subgroups remained significant even after adjusting for age and multiple hypothesis testing. The observed protective effects associated with the two aforementioned *CCL5* SNPs (rs3817655, rs2107538) may be attributed to a reduction in transcriptional activation, reduced protein levels, and ultimately reduced tumorigenic capacity. To our knowledge, there are no published reports on the impact of the *CCL5* rs3817655 SNP on prostate cancer susceptibility or its functional consequence on genes/proteins. On the other hand, the *CCL5* rs2107538 G-403A promoter SNP is associated with a decrease in protein expression detected in serum collected from Type II diabetic and disease-free subjects [16]. In addition, this locus has been evaluated in two independent prostate cancer studies. In a study involving 607 Caucasian male residents of Spain (297 cases, 3011 controls), Saenz-Lopez and co-workers (2008) observed a 1.44-fold increase in PCA risk among carriers of the *CCL5* rs2107538 GA+AA genotype (P = 0.039) [18]. However, this finding did not corroborate with a larger null report consisting of 1553 Caucasian men (i.e., 815 PCA cases, 738 controls) from Australia [33]. Our findings of a protective effect against PCA among our study participants is consistent with other published reports that reveal a decrease in the risk of developing gastric cancer, lymphoma, and type 1 diabetes [10,16,20]. Within a multi-ethnic pancreatic case–control study, the prevalence of the “A” allele was more frequent among disease-free Asian and African-Americans relative to pancreatic cases [23]. However, this allele was more prevalent among Caucasian pancreatic cases relative to controls. Collectively, these findings may suggest inheritance of genetic susceptibilities detected in the *CCL5* gene may vary across different racial/ethnic groups.

The functional impact of *CCL5* sequence variants is complicated by the high degree of linkage disequilibrium within both the promoter and intron 1 region. An and co-workers (2006), evaluated the impact of three SNPs detected in the promoter region (rs2280788 -28C/G, rs2107538 -405 G/A) and intron 1 of *CCL5* rs2280789 [17]. They demonstrated that transcriptional regulation of *CCL5* was primarily governed by an intron 1.1 A/G SNP (rs2280789). Intron 1.1 G allele corresponded with a strong decrease in transcriptional activity of RANTES; whereas, the -28G allele had a modest up-regulation in human cell lines. In our stratified analysis, the intron 1.1 *CCL5* rs2280789 AG or AG+GG genotypes were associated with a marginal 36-41% decrease in PCA risk among men of African descent from the US; however, these findings require further evaluation in a larger study set. The *CCL5* rs2280788-28 C/G SNP was not evaluated in the current study, since the C allele frequency is 0% for African-American men, as reported in NCBI. The functional consequence of *CCL5* SNPs is further perplexed by their interaction with downstream receptors.

The biological activity mediated by CCL5 is facilitated through its interaction with chemokine receptors (CCR1, CCR2, CCR3, and CCR5). However, relative to CCR1-3, CCR5 plays a more important role in CCL5-mediated cell migration [34]. CCR5, a member of the beta chemokine receptor family, is a seven transmembrane protein, which is expressed by T cells and macrophages. Over expression of CCR5 has been detected in aggressive prostate cancer tissue relative to benign prostatic hyperplasia [32]. The *CCR5* rs1799988 C allele is significantly associated with viral load set point (i.e., decreased time from asymptomatic HIV^+^ to AIDS and increased infectiousness) and AIDS progression [35]. However, there are no published reports for this 5’UTR SNP in relation to prostate cancer or other inflammatory/immune response-related diseases. In the current study, we observed a 1.52-1.75 fold increase in the risk of developing prostate cancer among all men of African descent who possessed the *CCR5* rs1799988 CC genotype. However, the impact of this SNP in relation to PCA risk was more pronounced among men of African descent from Jamaica relative to U.S. men. This increased risk may have an impact on transcriptional activity, which may result in increased protein levels of CCR5; however, this requires confirmation using *ex vivo, in vitro*, and micro-dissected tumor tissue-based assays.

The strengths, limitations and future directions of this study were considered. Forty-three sequence variants were evaluated in relation to prostate cancer risk among men of African descent from the U.S. and Jamaica. A strong correlation between the minor allele frequencies between these two study populations enabled us to pool genetic data to identify relationships that would have remained undetected if we evaluated the populations separately. As a result of pooling, we identified three SNPs (i.e., *CCL5* rs3817655, *CCL5* rs2107538, *CCR5* rs1799988) that were significantly associated with prostate cancer in the total population even after adjusting for age and multiple hypothesis testing. Upon stratification by study center, we cannot rule out the possibility that race/ethnic specific sequence variants may track with disease progression or prognosis. Consequently, future targeted sequencing will allow us to identify, evaluate and validate novel and commonly reported chemokine-associated SNPs as tumor classification and prognostication tools among African-American, Caribbean, African and European men. Such efforts will require pooled data from multi-center studies that seek to identify the genetic signatures related to prostate cancer health disparities domestically and globally. In addition, molecular biological studies are needed to understand the functional consequence of the *CCL5* and *CCR5* sequence variants on: mRNA expression/stability, or protein expression, structure and/or function. This will require well annotated observational studies that allow us to consider lifestyle, geographical, environmental, or cultural differences that may interact with genetic susceptibilities and subsequently modify PCA outcomes. In future studies, we will determine the impact of coding and non-coding SNPs on mRNA and protein levels in tissue collected from men of African and European descent using *ex vivo*, *in vitro*, and tissue-based assays. Lastly, study participants in the current study self-identified themselves as African-American, Caribbean, African, or Jamaican. Population admixture, which commonly occurs among men of African descent, may bias risk estimates. After adjusting risk estimates for West African Ancestry, risk estimates did not significantly vary when compared to unadjusted risk models (data not shown). In the current study, we had greater than 80% statistical power to observe effect sizes of ≥ 1.5 (or ≤ 0.67) and 1.55 (or ≤ 0.64) for the total and U.S. populations, respectively. All p-values in the current study were adjusted for multiple hypotheses testing to minimize false positive results using the false discovery rate. Despite these efforts, we cannot rule out the possibility that the significant relationships observed for the U.S. (*CCL5* rs3817655) and total populations (*CCL5* rs2107538, *CCL5* rs3817655, *CCR5* rs1799988) are suggestive and warrant further validation in larger studies. Future studies in our laboratory will focus on high-throughput targeted sequencing to evaluate the impact of novel and commonly reported CCL5 (rs2107538, *CCL5* rs3817655) and CCR5 (rs1799988) sequence variants on PCA susceptibility and disease prognosis among men of African descent. Even modest variations in genotype allele frequencies among men of African descent can reduce the chances of replicating single SNP effects within and between independent and validation study sets. To ensure reproducibility within future and ongoing studies, extreme care is needed to select study sets with comparable ancestry and genetic backgrounds.

## Conclusions

Our preliminary findings suggest that *CCL5* (rs2107538, rs3817655) and *CCR5* (rs1799988) SNPs may modify PCA susceptibility among men of African descent in the current study. In larger ongoing studies, we will evaluate the impact of individual or combination of chemokine-associated SNPs in relation to prostate cancer risk, tumor grade, biochemical or disease recurrence, and mortality. Such efforts will help to identify genetic markers capable of explaining disproportionately high prostate cancer incidence, mortality, and morbidity rates among men of African descent.

## Abbreviations

PCA: Prostate cancer; SNP: Single nucleotide polymorphism; CCL: Chemokine ligand; CCR: Chemokine receptor.

## Competing interests

LRK and KSK submitted a patent application for the main study findings mentioned in the manuscript. The authors declare that they have no competing interests.

## Authors’ contributions

LRK and KSK: conceptualized the project. LRK, KSK, CR, MJ, NM, MT, SM: participated in the study design. LRK: composed the manuscript. DJ, ER: revised subsequent manuscript drafts. DJ, NCK, SB, JER: conducted quality control and statistical analysis. LRK and GNB: co-supervised quality control analysis, data-management and statistical analysis. LRK, DJ, ER, KSK, SSB: interpreted the data, gave important intellectual input toward the introduction, results and/or discussion. All co-authors: read and edited the manuscript drafts as well as gave final approval of the final manuscript draft.

## Supplementary Material

Additional file 1 Baseline Characteristics among men of African Descent from the US. Click here for file

Additional file 2 Baseline Characteristics among men from Jamaica. Click here for file

Additional file 3 Functional Consequence of Chemokine-Associated Sequence Variants^a^. Click here for file
