# Supplementary material for: Chemokine Ligand 5 (CCL5) and chemokine receptor (CCR5) genetic variants and prostate cancer risk among men of African Descent: a case-control study
Source: Hered Cancer Clin Pract. 2012 Nov 20;10(1):16. doi: 10.1186/1897-4287-10-16 (PMC3527309; doi:10.1186/1897-4287-10-16)
Supplement: Additional file 1 — Baseline Characteristics among men of African Descent from the US. [file 1897-4287-10-16-S1.doc]

**Supplementary Material**

Additional File I. Baseline Characteristics among men of African Descent from

the US

| Characteristics | Cases | Controls | p value^a^ |
| --- | --- | --- | --- |
| Number of Participants, n | 170 | 433 | --- |
| Age at diagnosis (yrs), Median (range) | 65 (45-91) | 51 (27-89) | <0.0001 |
| Family History of Prostate Cancer, n (%)  Yes  No  Missing | 18 (16.7)  90 (83.3)  62 (36.5) | 9 (13.6)  57 (86.4)  367 (84.8) | 0.592 |
| PSA (ng/ml), median (range) | 7.0 (0.01-5,000) | 0.9 (0.0-3.9) | <0.0001 |
| PSA (ng/ml), n (%)  < 4  ≥ 4  Missing | 37 (23.1)  123 (76.9)  10 (5.9) | 416 (100.0)  0 (0.0)  17 (1.6) | <0.0001 |
| Gleason Score,^b^ n (%)  4  5  6  7  8  9  10  Missing | 12 (11.1)  14 (13.0)  29 (26.9)  32 (29.6)  5 (4.6)  12 (11.1)  4 (3.7)  62 (36.5) |  |  |
| Global WAA**,** mean (SD) | 0.79 (0.25-0.94) | 0.77 (0.25-0.94) | 0.107 |

Abbreviations: PSA, prostate specific antigen; ^a^Differences in frequencies were

tested by a Chi-square test of heterogeneity or Fisher’s Exact Test; differences in median

age (yrs) between cases and controls were tested using the Wilcoxon sum Rank test.
