# Supplementary material for: Chemokine Ligand 5 (CCL5) and chemokine receptor (CCR5) genetic variants and prostate cancer risk among men of African Descent: a case-control study
Source: Hered Cancer Clin Pract. 2012 Nov 20;10(1):16. doi: 10.1186/1897-4287-10-16 (PMC3527309; doi:10.1186/1897-4287-10-16)
Supplement: Additional file 2 — Baseline Characteristics among men from Jamaica. [file 1897-4287-10-16-S2.doc]

Additional File II. Baseline Characteristics among men from Jamaica

| Characteristics | Cases | Controls | p value^a^ |
| --- | --- | --- | --- |
| Number of Participants, n | 109 | 102 | --- |
| Age at diagnosis (yrs), Median (range) | 70 (49-80) | 60 (40-80) | <0.0001 |
| Family History of Prostate Cancer, n (%)  Yes  No  Missing | 17 (15.6)  92 (84.4)  0 (0.0) | 12 (11.8)  90 (88.2)  0 (0.0) | 0.272 |
| PSA (ng/ml), median (range) | 35.0 (4.0-10,000) | 1.2 (0.2-4.0) | <0.0001 |
| PSA (ng/ml), n (%)  < 4  ≥ 4  Missing | 0 (0.0)  108 (100.0)  0 (0.0) | 101 (99.0)  1 (1.0)  0 (0.0) | <0.0001 |
| Gleason Score,^b^ n (%)  4  5  6  7  8  9  10  Missing | ---  ---  45 (41.7)  38 (35.2)  13 (12.0)  10 (9.3)  2 (1.8)  1 (0.01) |  |  |

Abbreviations: PSA, prostate specific antigen; ^a^Differences in frequencies were

tested by a Chi-square test of heterogeneity or Fisher’s Exact Test; differences in median

age (yrs) between cases and controls were tested using the Wilcoxon sum Rank test.
