# Supplementary material for: Chemokine Ligand 5 (CCL5) and chemokine receptor (CCR5) genetic variants and prostate cancer risk among men of African Descent: a case-control study
Source: Hered Cancer Clin Pract. 2012 Nov 20;10(1):16. doi: 10.1186/1897-4287-10-16 (PMC3527309; doi:10.1186/1897-4287-10-16)
Supplement: Additional file 3 — Functional Consequence of Chemokine-Associated Sequence Variantsa. [file 1897-4287-10-16-S3.doc]

Additional File III. Functional Consequence of Chemokine-Associated Sequence Variants^a^.

| **dbSNP ID** | **Gene** | **Chr** | **Chr Position** | **Location** | **Nucleotide** | **Amino Acid Change** | **Predicted Functional Consequence** |
| --- | --- | --- | --- | --- | --- | --- | --- |
| rs1012656 | CCR6 | 6 | 167445293 | UTR-5 |  |  | TFBS |
| rs1024611 | CCL2 | 17 | 29603901 | 5' near gene |  |  | TFBS |
| rs1045879 | CXCR7 | 2 | 237154643 | Exon 1 | T>C | Leu266Leu |  |
| rs11076191 | CCL17 | 16 | 55997089 | Intron 1 |  |  | TFBS |
| rs11574914 | CCL21 | 9 | 34700338 | 5' near gene |  |  | TFBS |
| rs11574915 | CCL21 | 9 | 34700084 | UTR-5 |  |  | TFBS, Splicing |
| rs11574916 | CCL21 | 9 | 34699239 | UTR-3 |  |  | TFBS, miRNA |
| rs12721497 | CCR9 | 3 | 45918134 | Exon 3 | G>A | Met284Val | nsSNP, benign |
| *rs1488371* | *CCR9* | *3* | *45913093* | *Intron 2* |  |  |  |
| rs1556413 | CCR6 | 6 | 167444733 | 5' near gene |  |  | TFBS |
| rs17809012 | CCL7 | 17 | 29636557 | 5' near gene |  |  | TFBS |
| rs17880777 | CXCL12 | 10 | 44201208 | 5' near gene |  |  | TFBS |
| *rs1799987* | *CCR5* | *3* | *46386939* | *Intron 1* |  |  | *TFBS* |
| ***rs1799988*** | ***CCR5*** | ***3*** | ***46387263*** | ***UTR-5*** |  |  | ***TFBS*** |
| rs1800024 | CCR5 | 3 | 46387563 | Intron 2 |  |  | TFBS |
| rs1801157 | CXCL12 | 10 | 44188263 | UTR-3 |  |  | miRNA |
| rs2023305 | CCR6 | 6 | 167444888 | 5' near gene |  |  | TFBS |
| rs2032887 | CCL25 | 19 | 8027360 | Exon 3 | G>A | His101Arg | Splicing, probably damaging |
| **rs2107538** | **CCL5** | **17** | **31231893** | **5' near gene** |  |  | **TFBS** |
| rs2227010 | CCR5 | 3 | 46386546 | 5' near gene |  |  | TFBS |
| rs223895 | CCL17 | 16 | 55998397 | Intron 1 |  |  |  |
| rs2280789 | CCL5 | 17 | 31231116 | Intron 1 |  |  | TFBS |
| rs2282691 | CCL1 | 17 | 29712422 | Intron 2 |  |  |  |
| rs2286486 | CCR9 | 3 | 45902745 | 5' near gene |  |  | TFBS |
| rs2302004 | CCL24 | 7 | 75280791 | Intron 1 |  |  | TFBS |
| rs2302009 | CCL26 | 7 | 75236934 | UTR-3 |  |  | miRNA |
| rs266093 | CXCL12 | 10 | 44186214 | UTR-3 |  |  | miRNA |
| rs2812378 | CCL21 | 9 | 34700260 | 5' near gene |  |  | TFBS |
| rs2839685 | CXCL12 | 10 | 44201644 | 5' near gene |  |  | TFBS |
| rs2839695 | CXCL12 | 10 | 44193855 | UTR-3 |  |  | miRNA |
| rs3093023 | CCR6 | 6 | 167454280 | 5' near gene |  |  |  |
| rs3093024 | CCR6 | 6 | 167452783 | Intron 1 |  |  |  |
| ***rs3136685*** | ***CCR7*** | ***17*** | ***35973325*** | ***Intron 1*** |  |  |  |
| **rs3136687** | **CCR7** | **17** | **35971422** | **Intron 1** |  |  |  |
| **rs3817655** | **CCL5** | **17** | **31223754** | **Intron 2** |  |  | **TFBS** |
| rs41289608 | CCR9 | 3 | 45903142 | UTR-5 |  |  | TFBS, Splicing |
| rs4795896 | CCL11 | 17 | 29636365 | 5' near gene |  |  | TFBS |
| rs523604 | CXCR5 | 11 | 118260948 | Intron 1 |  |  |  |
| rs6550178 | CCR4 | 3 | 32968496 | Intron 1 |  |  | TFBS |
| rs7259568 | CCL25 | 19 | 8023608 | 5' near gene |  |  | TFBS |
| rs7559855 | CXCR7 | 2 | 237153189 | Intron 1 |  |  |  |
| rs7613548 | CCR9 | 3 | 45901573 | 5' near gene |  |  | TFBS |
| rs7632357 | CCR4 | 3 | 32967207 | 5' near gene |  |  | TFBS |

Abbreviations: Chr, chromosome; UTR, untranslated region; TFBS, transcription factor binding site, miRNA, micro RNA; nsSNP, nonsynonymous SNP; ^a^Forty-three chemokine-related SNPs were analyzed among men of African Descent. SNPs denoted in bold were significantly associated with PCA risk in the total population. Italicized SNPs were significantly associated with PCA risk in the Jamaican population. Columns shaded in gray symbolize SNPs that were significantly associated with PCA risk in the U.S. population.
